# Immunotherapeutic strategies for invasive bladder cancer: a comprehensive review

**DOI:** 10.3389/fimmu.2025.1591379

**Published:** 2025-04-30

**Authors:** Yingying Wang, Min He, Jian Li, Li Li

**Affiliations:** ^1^ Department of Pharmacy, The First People’s Hospital of Xiaoshan District, Hangzhou, China; ^2^ Department of Emergency, The First People’s Hospital of Xiaoshan District, Hangzhou, China

**Keywords:** bladder cancer, immunotherapy, PD-1/PD-L1 inhibitors, bladder-preserving therapy, neoadjuvant immunotherapy

## Abstract

Bladder cancer is a prevalent malignancy, with muscle-invasive bladder cancer (MIBC) presenting a significant therapeutic challenge. Standard treatments, including radical cystectomy (RC) and neoadjuvant chemotherapy, pose substantial risks and impact quality of life, leading to increasing interest in bladder-preserving therapies (BPT). Immunotherapy has revolutionized bladder cancer management, with strategies ranging from intravesical Bacillus Calmette-Guérin (BCG) to immune checkpoint inhibitors targeting programmed cell death protein 1 (PD-1) and its ligand (PD-L1). In BCG-unresponsive non-muscle-invasive bladder cancer (NMIBC), PD-1 inhibitors such as pembrolizumab offer promising response rates. In MIBC, neoadjuvant immunotherapy with agents like atezolizumab and pembrolizumab improves pathological complete response (pCR) and facilitates bladder preservation. Combination regimens integrating radiotherapy, chemotherapy, and immunotherapy not only enhance treatment efficacy but also exploit mechanisms such as immunogenic cell death and antigen release that further augment antitumor immune responses. This review provides a comprehensive analysis of current immunotherapeutic strategies for invasive bladder cancer, highlighting their clinical applications and future potential.

## 1 Introduction

Bladder cancer ranks among the most prevalent malignancies of the urinary system and is the tenth most commonly diagnosed cancer globally (1, 2). It is categorized into non-muscle-invasive bladder cancer (NMIBC) and muscle-invasive bladder cancer (MIBC) based on the depth of tumor invasion, with MIBC accounting for approximately 25% of cases and associated with a poorer prognosis (3). The standard treatment for MIBC involves neoadjuvant chemotherapy followed by radical cystectomy (RC) and pelvic lymph node dissection (PLND) (3, 4). However, RC is high-risk, especially for elderly patients with comorbidities, and is linked to perioperative mortality and complications like infections (5). This has driven interest in bladder-preserving therapies (BPT) (6). Studies show BPT achieves tumor control comparable to RC, with no significant difference in overall survival (OS) (7, 8), and 80% of BPT patients maintain an intact bladder five years post-treatment, with improved quality of life (9–12).

In recent years, immunotherapy has emerged as a transformative approach in cancer management (13), particularly through its ability to reprogram the tumor microenvironment (TME). Immune checkpoint inhibitors (ICIs) (e.g., PD-1/PD-L1 inhibitors) can reduce T-cell exhaustion, reshape cytokine networks, and modulate tumor-associated macrophages (TAMs) in ways that enhance immune-mediated tumor clearance (14, 15). These advancements have expanded the therapeutic landscape, providing hope for improved outcomes and quality of life for bladder cancer patients. This review aims to synthesize current research on immunotherapy for invasive bladder cancer, focusing on its role in bladder preservation and its integration into multimodal treatment strategies. We will describe the mechanisms of action, clinical efficacy, and future potential of immunotherapeutic agents, including intravesical BCG, immune checkpoint inhibitors, and novel combination therapies. By examining the latest advancements in immunotherapy, this review seeks to highlight its growing importance as a cornerstone in the management of bladder cancer, offering new avenues for personalized and effective treatment.

## 2 Immunotherapy for NMIBC

### 2.1 Intravesical BCG treatment

#### 2.1.1 Mechanism of action of BCG

BCG, an attenuated live strain of Mycobacterium bovis, was initially used for tuberculosis treatment and approved in the U.S. in 1990 for stage I bladder cancer. Intravesical BCG is the preferred treatment for preventing high-risk NMIBC recurrence and is endorsed by the European Association of Urology and the National Comprehensive Cancer Network (16). BCG’s therapeutic mechanism involves direct antitumor activity and immunomodulation (17). It adheres to the bladder wall, exerting cytotoxic effects, and induces an inflammatory response, recruiting immune cells. BCG interacts with fibronectin on tumor cells, prompting neutrophil recruitment. Neutrophils release TRAIL, inducing apoptosis (18). This dual mechanism underscores BCG’s unique role in NMIBC management, combining direct and immune-mediated antitumor actions.

#### 2.1.2 Factors influencing the efficacy of intravesical BCG therapy

Factors such as the strain of BCG, dosage, administration method, and the bladder microbiota significantly impact the efficacy of intravesical BCG therapy. Comparative studies reveal that, among various strains, the Russian and Connaught strains of BCG manifest a notably enhanced suppression of cell proliferation and cytokine responses in bladder cancer T24/J82 cells (19). Additionally, research indicates that patients with intermediate-risk NMIBC exhibit a more robust response to a full-dose BCG treatment over one year, whereas those with high-risk NMIBC benefit significantly from a three-year full-dose regimen (20). The local immune environment in cancer is also shaped by cytokine networks and immune cells (21–26), which can either promote or inhibit antitumor immunity (27–30). The bladder microbiota also plays a crucial role, potentially moderating mucosal inflammation by inhibiting pathways involving interleukins (IL-6, IL-8) and nuclear factor-kappa B (NF-kB), thereby influencing the BCG-dependent local inflammatory response essential for initiating immunotherapy. Preliminary findings suggest that pre-treatment with subcutaneous BCG injections prior to intravesical administration may enhance T-cell recruitment to the bladder and improve tissue responsiveness to BCG, correlating with increased recurrence-free survival rates and BCG-specific immunity (31).

#### 2.1.3 Mechanisms of resistance in intravesical BCG therapy

Understanding resistance mechanisms to intravesical BCG therapy is critical, particularly in refractory, resistant, or recurrent cases, often leading to radical cystectomy (32). A key mechanism involves PD-L1 upregulation on tumor cells, which interacts with PD-1 on T-cells, suppressing immune responses (33–35), and enabling cancer evasion (36–39). Biomarkers such as PD-L1 expression and Th1/Th2 polarization can help predict which patients are more likely to develop BCG resistance (40, 41). This has made PD-1/PD-L1 inhibitors a focal point in bladder cancer immunotherapy. Recent studies highlight the role of ICIs, such as PD-1/PD-L1, in cancer immune escape, with ICIs reactivating cytotoxic T cells to target tumors (42). Additionally, BCG may inhibit TNF-α-mediated p53 expression, reducing apoptosis (43–45). Combining BCG therapy with PD-L1 inhibitors (intravesical or systemic) or TNF-α could overcome resistance, prolong BCG response, prevent recurrence, reduce BCG dosage, and minimize adverse effects.

#### 2.1.4 BCG and IL-2 combined immunotherapy

Recent research has focused on enhancing the efficacy of BCG therapy and reducing the required dosage through combination treatments, which may improve prognosis and the safety of tumor immunotherapy, thereby reducing the incidence of adverse events leading to the discontinuation of BCG therapy. Steinberg (46) studied 52 high-risk NMIBC patients who had experienced at least one failure of previous BCG therapy and underwent a six-cycle regimen of quadruple immunotherapy (intravesical BCG + interferon + IL-2 + subcutaneous granulocyte-macrophage colony-stimulating factor). Among these patients, 55% remained recurrence-free at one year, and 53% at two years, thus avoiding cystectomy despite 6% being unable to tolerate the full induction.

### 2.2 Keyhole limpet hemocyanin and MCNA

KLH functions as an immunotherapeutic agent by eliciting a robust systemic immune response specifically targeting bladder cancer cells. This mechanism involves the induction of high titers of tumor-specific immunoglobulin G antibodies. Lammers et al. (47) conducted a pivotal prospective randomized Phase III clinical trial comparing the therapeutic efficacy and safety profile of KLH versus mitomycin in intermediate and high-risk non-muscle invasive bladder cancer (NMIBC) patients. The study demonstrated superior clinical outcomes in the KLH treatment arm, with a significantly lower tumor recurrence rate of 34% compared to 61% in the mitomycin group, indicating KLH’s potential as a more effective therapeutic option for this patient population. The Mycobacterium phlei cell wall-nucleic acid complex (MCNA) contains antigens derived from the cell wall of Mycobacterium phlei attached to a mixture of nucleic acid oligomers (48). Morales (49) performed a Phase II single-arm study to evaluate the efficacy and safety of MCNA in 129 patients with NMIBC who had failed BCG therapy. The study outcomes indicated that the one-year, two-year, and three-year progression-free survival rates were 87.3%, 79.8%, and 77.7% respectively, with a two-year overall disease-free survival rate of 19%. The patients tolerated MCNA well, making it an important option for bladder preservation.

### 2.3 PD-1 and PD-L1 inhibitors

Currently approved PD-1 inhibitors for the treatment of bladder cancer include pembrolizumab and nivolumab. Pembrolizumab exerts its antitumor immunotherapeutic effects by inhibiting the PD-1/PD-L1 interaction. A clinical trial involving 370 patients with advanced or metastatic urothelial carcinoma demonstrated that pembrolizumab achieved an objective response rate of 29% (50), with 7% of patients reaching complete remission. Nivolumab selectively blocks the interaction between PD-1 and PD-L1/Programmed Death-Ligand 2 expressed on tumor cells, disrupting PD-L1-mediated signaling and thereby restoring the antitumor response of effector T cells (51). Clinical trial data show that nivolumab treated patients with metastatic urothelial carcinoma had an overall objective response rate of 19.6% and a complete remission rate of 2% (52). Approved PD-L1 inhibitors in bladder cancer include atezolizumab, durvalumab, and avelumab. Atezolizumab demonstrated an objective response rate of 23% in metastatic urothelial carcinoma (53). Durvalumab, an immunoglobulin G monoclonal antibody, operates by obstructing the PD-1/PD-L1 interaction, thus enabling T cells to recognize and eliminate tumor cells. A clinical trial demonstrated that durvalumab treatment in patients with urothelial carcinoma achieved an overall objective response rate of 17.8%, and this increased to 27.6% in patients with high PD-L1 expression (54). Avelumab, an anti-PD-L1 immunoglobulin G1 monoclonal antibody, selectively blocks the PD-1/PD-L1 interaction. It reached an 18.2% overall response rate, with up to 53.8% in high PD-L1–expressing patients (55) (**Table 1**).

**Table 1:** Immunotherapy strategies for NMIBC.

Therapy	Mechanism of Action	Efficacy	Clinical Outcomes
BCG	Direct antitumor activity, immune modulation, induces inflammatory response, cytotoxic effects	Effective in preventing high-risk NMIBC recurrence	Recruits immune cells, induces apoptosis via TRAIL from neutrophils
PD-1 Inhibitors	Inhibits PD-1/PD-L1 interaction, restoring antitumor response of T cells	Variable efficacy, with pembrolizumab achieving a response rate of 29%	Approved for advanced or metastatic urothelial carcinoma
PD-L1 Inhibitors	Blocks PD-L1, enhancing T cell-mediated tumor cell destruction	Variable, atezolizumab achieved a response rate of 23%	Approved for treatment of bladder cancer with specific response rates in trials
KLH	Elicits systemic immune response, presensitization required	Lower recurrence rate compared to mitomycin	Induces high titers of immunoglobulin G against bladder cancer cells
MCNA	Contains antigens from Mycobacterium phlei, attached to nucleic acid oligomers	Progression-free survival rates at 1, 2, and 3 years were 87.3%, 79.8%, and 77.7%, respectively	Well tolerated with significant bladder preservation effects

## 3 Immunotherapy for MIBC

### 3.1 Bladder-preserving treatment options and advantageous populations

Currently, a variety of BPTs are available, such as tri-modality therapy (TMT), maximal transurethral resection of bladder tumor (mTURBT) combined with chemotherapy and/or radiotherapy, partial cystectomy (PC) combined with chemotherapy and/or radiotherapy, and integrative immunotherapy-based approaches. The implementation of BPTs requires multidisciplinary consultation and rigorous evaluation. Patients considered to be particularly suited for BPTs meet the following criteria: (1) Patient factors: high compliance, good bladder function and capacity; and (2) Tumor factors: stage T2, solitary tumor, absence of concomitant carcinoma *in situ*, absence of hydronephrosis, and complete TURBT (56). For high-risk patients, such as those where TURBT cannot achieve complete tumor removal, widespread carcinoma *in situ*, presence of hydronephrosis, diffuse tumors, and stage cT3-4, TMT remains a viable option, although the likelihood of cure is significantly reduced (57). Studies have demonstrated that immunotherapy can markedly downstage tumors, potentially converting non-ideal candidates into suitable ones for bladder preservation (4).

### 3.2 The Role of immunotherapy in bladder preservation for patients with BCG-unresponsive MIBC

National guidelines strongly recommend one year of BCG bladder instillation therapy post-TURBT for high-risk MIBC. However, approximately 30% of patients experience tumor recurrence, 10% progress (58), and 50% do not respond to BCG (59). Adverse reactions like fever, cystitis, and hematuria further limit tolerability. For BCG-unresponsive cases, RC is the primary guideline-recommended treatment, though immunotherapy has emerged as a bladder-preserving alternative (60). Studies show Pembrolizumab monotherapy achieves a 41% complete response (CR) rate at three months in BCG-unresponsive high-risk MIBC patients refusing RC, with 80% maintaining CR for over six months and a median remission duration of 16.2 months (61). These results led to FDA approval of Pembrolizumab for such patients in January 2020. Similarly, Atezolizumab monotherapy reported CR rates of 41.1% at three months and 26.0% at six months in BCG-unresponsive carcinoma *in situ* patients, with an 83.6% adverse event rate (62).

Clinical trials have further validated the role of ICIs in NMIBC and MIBC settings. The KEYNOTE-057 trial updated results indicated durable CR rates in high-risk BCG-unresponsive NMIBC treated with pembrolizumab, reinforcing its approval status (61). Additionally, the CheckMate 274 trial in the adjuvant setting for MIBC demonstrated that nivolumab significantly improved disease-free survival compared with placebo in patients at high risk of recurrence post-RC (63). Inflammatory factors are pivotal in disease’s progression (64–67). Innovative immunotherapies like the IL-15 superagonist N-803 have shown promise, with a 92% bladder preservation rate at 12 months and 99.5% tumor-specific survival at 24 months, alongside a low grade 3+ adverse event rate of 3% (68). Additionally, the antibody-drug conjugate vicinium achieved a 40% CR rate at three months in carcinoma *in situ* patients, with a 96% two-year overall survival rate (69). These advancements highlight the potential of novel immunotherapies as effective alternatives for BCG-unresponsive high-risk NMIBC patients, offering viable bladder-preserving options as clinical trials progress.

### 3.3 Applications of immunotherapy in neoadjuvant therapy prior to bladder-sparing surgery

MIBC constitutes a lethal malignancy with a five-year survival rate of 50%guidelines universally recommend a neoadjuvant chemotherapy regimen based on cisplatin (70), followed by RC. However, given the significant decline in quality-of-life post-RC, a subset of patients strongly prefers bladder preservation. A critical concern with BPT is their ability to achieve survival outcomes comparable to those of current RC protocols. The pursuit of bladder preservation without compromising prognosis has emerged as a focal area of research. The application of single-agent or combination immunotherapeutic agents as neoadjuvant interventions has enhanced the rates of pathological complete response (pCR) and downstaging in MIBC patients (71). Compared to chemotherapy, the majority of adverse reactions associated with immunotherapy are mild (grades 1–2), offering a safer and more effective treatment modality for elderly and frail MIBC patients. Neoadjuvant immunotherapy has expanded the possibility of bladder preservation for a broader cohort of MIBC patients (71).

The following delineates several immunotherapy-based neoadjuvant bladder preservation strategies: (I) Single-agent immunotherapy neoadjuvant bladder-preserving protocol: To benefit patient intolerant to chemotherapy, a multicenter phase II clinical trial on Atezolizumab (ABACUS study) demonstrated a pCR rate of 31% across the general population, with a one-year tumor recurrence-free survival (RFS) of 79%. In PD-L1 positive patients, the pCR rate reached 37%, with 54% experiencing downstaging to NMIBC, and a one-year RFS of 75% (72). Another phase II clinical trial using Pembrolizumab (PURE-01 study) reported a pCR rate of 37%, with 55% of patients achieving tumor downstaging (73). (II) Combination therapy and chemotherapy in neoadjuvant bladder-preserving protocols: The GC (gemcitabine + cisplatin) regimen remains the standard chemotherapy for bladder cancer and the conventional neoadjuvant therapy for MIBC. However, recent studies show improved efficacy with immunotherapeutic agents: Pembrolizumab combined with GC achieved a 44.4% pCR rate (74); Nivolumab with GC reached a 48.0% cCR rate, with cCR patients showing one-year survival and metastasis-free rates of 100.0% and 81.2%, respectively, and 78.0% maintaining bladder preservation (75). These combination regimens harness immunogenic cell death induced by chemotherapy, enhancing tumor antigen presentation and T-cell priming. The synergy between chemotherapeutic agents and ICIs potentially increases tumor immunogenicity, resulting in improved response rates (76). While single-agent immunotherapy may be safer in cisplatin-intolerant populations, combination strategies could yield more robust pCR and downstaging rates. Long-term follow-up data, ideally from randomized controlled trials, are needed to confirm survival benefits.

### 3.4 Application of immunotherapy in the adjuvant comprehensive treatment following bladder-sparing surgery

Current clinical guidelines universally endorse the implementation of rigorous adjuvant chemotherapy and/or radiotherapy for MIBC patients who have been meticulously selected and assessed following PC or mTURBT procedures (4, 77). Radiotherapy can release tumor-specific antigens, facilitate T-cell priming, and increase MHC-I expression on tumor cells, thereby amplifying the effects of ICIs. For cisplatin-ineligible MIBC patients, radiotherapy plus durvalumab yielded a one-year progression-free survival (PFS) rate of 73.0% and an OS rate of 83.8% (78). Another protocol combining radiotherapy with durvalumab and tremelimumab reported a CR rate of 81% (48), demonstrating the synergistic potential of multimodal regimens (79). TMT is a conventional bladder-preserving adjuvant treatment, which emphasizes the synchronous application of chemotherapy and radiotherapy. Ongoing clinical trials investigating TMT combined with immunotherapy have reported encouraging results. For instance, intermediate trial outcomes for MIBC patients undergoing mTURBT followed by Pembrolizumab combined with concurrent chemoradiotherapy revealed a cCR rate of 90%, with final study results highly anticipated (80–82). As immunotherapy continues to evolve, combining TMT with ICIs could increase cCR rates and further improve bladder preservation. These synergistic effects, coupled with a generally favorable safety profile, position immunotherapy as a key component of future bladder preservation strategies (**Table 2**).

**Table 2:** Immunotherapy for bladder preservation in MIBC.

Treatment Method	Description	Pathological Complete Response (pCR)	Survival Outcomes
Single-agent immunotherapy	Use of agents like atezolizumab and pembrolizumab as a neoadjuvant treatment	Rates of 31% for atezolizumab, 37% for pembrolizumab	Enhanced survival and downstaging in MIBC patients
Combination therapy (chemo + immunotherapy)	Pembrolizumab or nivolumab combined with GC (gemcitabine + cisplatin)	44.4% with pembrolizumab + GC, 48.0% with nivolumab + GC	High one-year survival and metastasis-free rates
Radiotherapy combined with Immunotherapy	Radiotherapy with durvalumab or durvalumab + tremelimumab	73.0% PFS with durvalumab, 81% CR with dual immunotherapy	High overall survival rates
TMT combined with Immunotherapy	Trimodal therapy integrating chemotherapy, radiotherapy, and immunotherapy	90% clinical complete response in interim trials	Comparable to radical cystectomy with potential quality of life improvements

## 4 Conclusion

Immunotherapy is transforming the management of invasive bladder cancer by offering viable alternatives to standard radical cystectomy, particularly for patients with comorbidities or those seeking bladder preservation. Intravesical BCG remains essential for high-risk NMIBC, but its limitations and resistance patterns highlight the growing role of ICIs such as pembrolizumab, nivolumab, atezolizumab, durvalumab, and avelumab. In MIBC, neoadjuvant and adjuvant immunotherapy, especially when combined with chemotherapy or radiotherapy, can enhance pathological complete response and long-term survival, facilitating bladder preservation without sacrificing oncologic outcomes. Looking forward, biomarker-driven clinical trials are urgently needed to personalize immunotherapy and identify patients most likely to benefit; overcoming primary and acquired resistance to ICIs remains a crucial challenge, spurring research into novel agents and combination approaches; and optimizing treatment sequencing, particularly how best to integrate immunotherapy with chemo- and radiotherapy, will be pivotal for advancing the standard of care. As research expands our understanding of the TME, immunotherapy is poised to become an even more integral component of bladder cancer management, ultimately improving patient outcomes and quality of life.
